# Responsible Use of Pop Culture and Communication in the Face of Ebola Virus

**DOI:** 10.1371/journal.pntd.0003890

**Published:** 2015-08-06

**Authors:** Brandon Brown, Melissa Nasiruddin, Alexander Dao, Monique Halabi

**Affiliations:** 1 University of California, Irvine Program in Public Health, Department of Population Health and Disease Prevention, Irvine, California, United States of America; 2 Midwestern University, Arizona College of Osteopathic Medicine, Glendale, Arizona, United States of America; 3 A.T. Still University, School of Osteopathic Medicine, Mesa, Arizona, United States of America; 4 California State University Los Angeles, School of Nursing, Los Angeles, California, United States of America; Common Heritage Foundation, NIGERIA

## Introduction

Tense music and flashy title cards on the evening news instill a sense of impending doom as we learn that the number of persons infected by Ebola is estimated to reach the millions in a matter of weeks [1]. Disheveled scientists in white coats spit microbiological jargon like it is poetry, hastening down laboratory hallways with desperate and bemused political leaders in tow. Their conversation continues offscreen, over a montage of men clad in hazmat suits scrambling to treat patients while their families wait, fearing the worst. A Hail Mary of a scientific breakthrough suddenly surfaces—a vaccine that still needs testing, prototype nanobots, or new rapid testing methods to battle the widespread infection. The audience awaits a miracle.

This exposition echoes both archetypes of Hollywood and the media’s response to epidemics around the world. Though hidden deep in the flash and pop of panic-inducing graphics, there is valuable information that can be used for educational purposes. The ease of access to popular culture, media, and technology offers the opportunity to benefit from Ebola’s pervasive presence through dissemination of correct and useful information both locally and across the globe.

According to the Centers for Disease Control and Prevention (CDC), the current outbreak of Ebola is one of the largest in recorded history, having spread across Liberia, Guinea, and Sierra Leone [2]. A small number of imported cases were also reported in Spain, Italy, the United States, other western African nations, and as of December 2014, the United Kingdom; most of these imported cases were health care workers who had been exposed to the pathogen while providing aid to the stricken parts of western Africa [2,3]. The CDC reports, as of May 26, 2015, that Ebola has infected at least 27,000 people and killed over 11,000 individuals worldwide [2].

Social media continues to develop as a mainstream reference point for sharing and discussing current events. Its role as a platform for discourse has been noticeably growing in the wake of the Ebola epidemic [4]. Prior studies emphasize the use of multiple modes of communication in order to maintain the propagation of correct information to broad populations [5,6]. Despite the low general knowledge of Ebola in the US, there are avenues of information dissemination that can be more quickly utilized to impact a wider audience. In order to provide accurate, consistent information, our leading health-focused institutions should bolster their use of technology and apply popular culture to reach and inform a greater portion of the public.

## Current Use of Pop Culture in the Ebola Epidemic

The CDC, the World Health Organization (WHO), and other public health agencies have leveraged popular social media to distribute up-to-date and accurate information on Ebola [7,8], while social websites such as Reddit, to which users can submit content organized by areas of interest, allow for the assimilation of unverified information [9]. A literature review on the use of social networking sites for influencing health behavior demonstrated a particularly valuable aspect of social media: its cost-effective ability to reach hard-to-reach underserved and minority populations [10]. Health information reaches consumers at various levels that differ based on demographic and socioeconomic factors. For example, data from the Health Information National Trends Survey found that individuals who sought out health information tended to have regular health care access and were more likely to earn over US$50,000 in income; conversely, males, people older than 65 years of age, and people identifying as Hispanic were less likely to seek out that information [11]. Because of differences in how these groups receive, trust, and process health information, mechanisms by which pertinent health information is disseminated must be diversified in order to maximize the audience reached. This can be done through media accessed by members of multiple socioeconomic and cultural strata. Examples of useful media include film, books, pamphlets, the Internet, and crowdsource mapping, among others. Smart phone apps and health reminders through text messages are some ways that technology has been used to help raise awareness about the epidemic [12,13], but there are still other ways that social media and popular culture can be used to further spread vital public health information. The public is eager for this information, as clearly demonstrated with the success of television programs such as the Dr. Oz show in promoting information-seeking behavior [14].

## Demonstrated Advantages of Pop Culture and Diverse Technology in Public Health Campaigns

Aside from the ability to triage accurate information, pop culture and technology flaunt remarkable education and fundraising potential. The “ALS [amyotrophic lateral sclerosis] ice bucket challenge” that permeated social media in 2014 produced over a 3,500% increase of donations in a single month, as compared to the previous year [15]. Pop-culture icons such as superheroes and zombies shine as tools for education and discussion of complex scientific concepts. Their use establishes a middle ground between scientists and the general public, placing scientific concepts in a comfortable and familiar mental landscape [16–18]. Additionally, the wide reach of technology makes it possible to recruit more help in advancing knowledge through crowd sourcing for research and science projects. The Health Maps website and the Humanitarian OpenStreetMap Team, for example, have recently begun collaborating with humanitarian agencies such as Doctors Without Borders and the Red Cross to create outbreak maps that benefit aid workers in addressing disease outbreaks [19–21]. This form of crowdsourcing and other technologically based intervention efforts could be modified for Ebola.

## Previous Examples of Pop Culture in Public Health Campaigns

In the past, different audiences have been reached through increasingly creative avenues [22]. For example, Delta Airlines is currently on their sixth in-flight safety video that uses humor to deliver their low-interest safety message [23]. Comedy helps the airline teach safety information to an otherwise unenthusiastic group of passengers, capturing their attention with a variety of humorous examples in the videos. Using pop culture in the delivery of pertinent Ebola information may help pique the interest of previously uninformed individuals, prompting them to follow the outbreak in a manner that shadows lightness over a very serious health issue, making it easier to absorb. This is not to say that these light-hearted messages should downplay the seriousness of the problems; rather, they should ease the digestibility but never overshadow the true gravity of the issues themselves. Such tactics ought to be verified for tact and integrity before being disseminated to the public.

Films such as 2011’s *Contagion* have also been useful in teaching the public about how local health departments and the CDC respond to crises, and in some ways it has even impacted how health scientists respond to and work to prevent outbreaks [24]. Ultraviolet (UV)-light-producing robots are becoming popular in hospital room sterilization [25,26], and the advent of smartphones allows consumers to purchase game applications that allow them to design and observe the virulence and transmission patterns of fictional pathogens [27]. These hyperboles of film are quickly becoming commonplace.

Another example of the use of familiar, relatable subject matter to disperse relevant public health and safety information was the CDC’s introduction of zombies into the public health realm in mid-2011. Following the catastrophic 2011 Tohoku earthquake in Japan, the CDC created a dramatized public health bulletin to prepare for a zombie apocalypse, in an effort to draw readers in and bolster awareness for the importance of disaster preparedness [28]. The authors of the educational bulletin used the popular media phenomenon of a zombie apocalypse as a valuable teaching tool to reach audiences that they may not have reached otherwise.

## Limitations of Diverse Technology/Pop Culture in Public Health Campaigns

Although the instantaneous dispersal of information through social media is one of the advantages to using pop culture to spread relevant public health information, it may also be seen as a double-edged sword. The rapidity of information release allows for the chance of spreading incorrect or misleading information, which can be seen in the media frenzy regarding the Ebola outbreak in West Africa and the resulting panic surrounding isolated cases of Ebola in the US [29]. With social media, text messaging, phone calls, radio, and television, there are many avenues of information collection that allow for the rapid dissemination of misinformation. The consequences of this were seen in late 2014 in Nigeria, when a prescription for a salt-water bath for Ebola prevention quickly spread through social media, eventually encouraging people to drink excessive amounts of salt water and leading to a few reported deaths [30,31]. Another limitation relates to the idea that organized sources of social media may provide information only to the technologically literate individuals that seek this information, potentially alienating a substantial segment of the public that are impacted by these public health issues.

## Conclusion and Recommendations

Rather than frighten the public through scattered, gloomy newscasts about new Ebola cases, well-known popular culture tools should be used to disperse relevant health precaution information to a greater target audience using an entertaining, more recognizable form. Many consumers prefer to take initiative and seek desired information on their own by turning to the Internet for health information, with nearly 75% of Internet users searching for information about health in the past year [32]. Popular culture can be used to educate mass populations about important information regarding the Ebola outbreak, being used as a hook to draw audiences in and as a familiar vehicle for information dissemination. With the fifth season of the popular zombie series *The Walking Dead* recently premiering (15 million viewers in February 2015), this is an ideal time to use this popular culture tool in relation to the Ebola outbreak, whether it be through social media websites, large-scale public health website bulletins, commercials, or other outlets. Those professionals in charge of disseminating information must consistently be on the lookout for what characters are popular in the current time, and they must be creative in how these icons can be related to a disease or outbreak.
